# The Detection of Lymphatic Invasion in Colorectal Polyp Cancer Using D2-40 Immunohistochemistry and Its Association With Prognosis

**DOI:** 10.7759/cureus.11394

**Published:** 2020-11-09

**Authors:** Mohammad R Goodarzi, David Mansouri, Andrew C Kidd, Clare Orange, Fraser Duthie

**Affiliations:** 1 Wolfson Medical School, University of Glasgow, Glasgow, GBR; 2 Department of Colorectal Surgery, Glasgow Royal Infirmary, Glasgow, GBR; 3 College of Medical Veterinary and Life Sciences, University of Glasgow, Glasgow, GBR; 4 Department of Pathology, Queen Elizabeth University Hospital, Glasgow, GBR

**Keywords:** immunohistochemistry staining, colon cancer and colon polyps, lymphatic spread

## Abstract

Introduction

The aim of this study was to compare the detection of lymphatic invasion using haematoxylin and eosin (H&E) staining versus D2-40 immunostaining on specimens from a retrospective cohort of patients with colorectal polyp cancer and to investigate the association of lymphatic invasion, detected by either method, with survival.

Methods

Specimens from patients with pathologically diagnosed colorectal polyp cancer were selected from the Greater Glasgow and Clyde Bowel Cancer Screening Registry for D2-40 immunohistochemistry staining. Clinicopathological information was retrieved from patient electronic records including analysis of pathology reports to determine if a lymphatic invasion was detected using H&E staining.

Results

Over 100 patients were included in this study with a median age at polypectomy of 66 years (range 50-76). All patients were followed up for a minimum of four years and five patients died due to colorectal cancer. The lymphatic invasion was detected in 8% of cases by H&E staining and 23% of cases with D2-40 immunostaining. Only D2-40-detected lymphatic invasion showed a statistically significant relationship with colorectal cancer-specific mortality using univariate analysis (p=0.01). Survival analysis performed separately by Cox regression demonstrated that lymphatic invasion detected by D2-40 immunostaining was associated with worse disease-specific survival (hazard ratio [HR] 14.07, 95% CI 1.57-125.97, p=0.018).

Conclusion

This study shows that D2-40 immunostaining can improve the detection of lymphatic invasion in colorectal polyp cancer when compared to H&E staining. In addition, the lymphatic invasion detected by D2-40 immunostaining significantly associates with survival allowing it to be used as a prognostic indicator in colorectal polyp cancer.

## Introduction

Screening for colorectal cancer by means of faecal occult blood testing (FOBt) has been offered in the United Kingdom since 2006 to individuals aged between 50 and 74 years old. Most colorectal polyps that are detected and removed as a result of screening contain benign adenomas [1]. However, 0.8-5.6% of all colorectal polyps and up to 10% of polyps removed as part of screening contain adenocarcinomas and are referred to as colorectal polyp cancers [2,3]. Similar to all early adenocarcinomas, the risk of metastasis is low. Nevertheless, this risk is not negligible and a small subset of patients with polyp cancer develop metastatic disease, the principle cause of colorectal cancer mortality [4]. 

A greater understanding of the prognostic characteristics associated with colorectal polyp cancers is required to allow improved risk stratification of patients so that high-risk individuals can be considered for further surgical interventions following polypectomy. Currently, the most widely used prognostic indicator in polyp cancer is the depth of invasion; determined by Kikuchi levels for sessile lesion [5] or Haggitt’s levels for polypoid lesions [6]. However, these scoring systems were described using resection specimens and thus are difficult to apply in polypectomy specimens which often contain neither muscularis propria (required for Kikuchi scoring) nor Haggitt’s level 4 [7].

Other prognostic indicators used in colorectal polyp cancer include: resection margin status, tumour differentiation, tumour budding, and lymphovascular invasion (LVI) [8,9]. LVI has been shown to be a powerful prognostic indicator in all stages of colorectal cancer [10-13]. Patients with primary colorectal cancer demonstrating LVI are more likely to have aggressive disease, nodal metastasis, and worse five-year overall and five-year disease-free survival [14]. The detection of LVI relies on detecting tumour cell invasion into either blood vessels or lymphatic channels. Elastica staining is used to highlight the elastic membranes of blood vessels to aid the detection of venous invasion [15]. However, no analogous staining procedure is routinely used to highlight lymphatic channels. Therefore, detection of lymphatic invasion in colorectal cancer relies on standard haematoxylin and eosin (H&E) staining which is associated with low sensitivity and poor accuracy [16]. 

D2-40 is a monoclonal antibody that specifically binds to podoplanin, a 38 kDa transmembrane glycoprotein, that is highly expressed in lymphatic endothelium [17]. D2-40 does not cross-react with vascular endothelium and thus is a selective marker of lymphatic vessels during immunohistochemistry staining [18]. D2-40 immunostaining has been reported to improve the detection of lymphatic invasion in primary cancers of the breast, skin, prostate, cervix, and colon [19]. Reports have also shown that lymphatic invasion detected by D2-40 has a prognostic value in stage I, stage II, and all stages of colorectal cancer [16,20,21]. However, the use of D2-40 immunostaining has never been investigated specifically in colorectal polyp cancer, a disease in which there is a demand for better prognostication. 

The aim of this study was to compare the detection rates of lymphatic invasion between standard clinical practice using H&E staining and the novel D2-40 immunostaining technique in a retrospective cohort of patients with colorectal polyp cancer. The association of lymphatic invasion with disease-specific survival, detected by either H&E or D2-40 staining, was investigated and compared to assess the prognostic value of either technique. The prognostic significance of other clinicopathological variables was also evaluated. All results are reported according to the REMARK criteria for recommending new prognostic tumour markers.

## Materials and methods

Patients were selected from the Greater Glasgow and Clyde Bowel Cancer Screening Registry from April 2009 to March 2011. This registry contains information on all individuals living within the Greater Glasgow area invited to FOBt screening. Inclusion criteria were set as patients who had pathologically diagnosed colorectal polyp cancer on colonoscopy after positive FOBt. Colorectal polyp cancer was defined according to the pT1 criterion of the American Joint Committee on Cancer (AJCC) TNM staging system as any sessile or polypoid lesion containing colorectal cancer “showing invasion through the muscularis mucosae into the submucosa but not beyond” (p.115) [22]. 

All tissue processing, staining, and inspection were performed within the Glasgow University Pathology Unit of the Queen Elizabeth University Hospital. All archived diagnostic H&E stained slides were initially inspected to identify specimens containing polyp cancers. The corresponding formalin-fixed and paraffin-embedded blocks for these cancer-containing slides were requested from the NHS Greater Glasgow and Clyde Biorepository. Once selected, the appropriate specimens were sectioned at 2.5 µm thick by a Leica RM2245 microtome, and sections were floated onto a water bath set at 40 °C before adhering to slides. 

Simultaneous dewaxing and antigen retrieval of slides was performed using heat-induced epitope retrieval in a Lab Vision PT Module (Thermo Fisher Scientific) for 30 minutes at 97 °C in EDTA buffer solution at pH 8. After removal, slides were left to cool in distilled water at room temperature for 10 minutes before both protein and peroxidase blocking procedures were performed. Samples were incubated in Lab Vision Ultra Block (Thermo Fisher Scientific, Waltham, USA) for five minutes to reduce nonspecific background staining followed by incubation in 2% hydrogen peroxide for five minutes to quench any intrinsic peroxidase activity. Primary antibody exposure followed with monoclonal mouse D2-40 antibody (Dako Product #M3619) diluted with antibody diluent (Dako Product #S0809) to a dilution of 1:100. During immunohistochemistry optimisation, 1:50 and 1:200 solutions were also tested but 1:100 dilution was used for all patient specimens as it provided optimal antibody detection. The diluted primary antibody was applied to slides for 30 minutes at room temperature, before Primary Antibody Amplifier Quanto (Thermo Catalogue #TL-060-QPB) and HRP Polymer Quanto (Thermo Catalogue #TL-060-QPH) were applied separately to the slides for 10 minutes each as secondary and tertiary amplification steps, respectively. 

The slides were washed with distilled water and then the chromogenic reaction was induced by adding DAB Quanto Chromogen (Thermo Catalogue #TA-002-QHCX) to each slide for five minutes to allow visualisation of the immunostaining. Nuclear counterstaining was performed by applying Haematoxylin Z (CellPath Catalogue #RBA-4201-00A, Newtown, UK) to all slides for 3 minutes followed by rinsing of slides in tap water, 1% acid alcohol solution (Genta Medical Catalogue #AAL050, York, UK), and Scott’s tap water substitute (Leica Biosystems #3802901E, Wetzlar, Germany). Dehydration of slides was carried out using increasing concentrations of alcohol (70%, 90%, 100%) and 100% xylene solution. The stained slides were finally sealed by mounting a coverslip. 

Immunohistochemistry optimisation and confirmation of staining specificity were validated by simultaneously performing the D2-40 immunostaining process for positive control tissues derived from tonsil, skin, and colon. Each batch of D2-40 staining included a separate negative control slide in which the mouse IgG1 antibody was used, instead of D2-40. In addition to these controls, each stained slide acted as its own internal control as during inspection it was ensured that only the lymphatic channels were stained. No further confirmation of the specificity of antigen-binding was required as a diagnostic grade of the D2-40 antibody (with previous Western blot testing) was used. 

The assessment of lymphatic invasion was performed using the recommendations set by the Japanese Society for Cancer of the Colon and Rectum (JSSCR) by viewing all sides at 4× magnification to identify suspected lesions and assessing these further at higher magnifications [23]. The presence of a positively stained endothelial cell monolayer with a lumen was identified as a lymphatic vessel in D2-40 stained slides and lymphatic invasion was defined as the invasion of at least one tumour cell into a lymphatic vessel [24,25]. Every slide was inspected by the lead investigator and an expert senior pathologist to confirm the detection of lymphatic invasion. Both these individuals were blinded to the retrospective clinical and pathological outcomes until the end of the study. 

The patient’s electronic records were examined by another investigator blinded to the results of D2-40 immunohistochemistry staining. The clinical data gathered on each patient included: location of the removed polyp, if the patient had undergone further resectional surgery, and if applicable, date of death and cause of death. Pathological data - including whether there was the detection of lymphatic invasion - were also collated from the diagnostic pathology reports of each patient’s polypectomy by a blinded investigator. The detection of lymphatic invasion in these reports was used to represent H&E-detected lymphatic invasion. The reported grades of the tumours were also recorded into two categories: (i) poor or (ii) moderate and well-differentiated. 

The Shapiro-Wilk test was used to assess for normality of data. The difference between proportions of lymphatic invasion detected by H&E staining and D2-40 immunostaining was calculated using McNemar's test with the paired confidence interval. Univariate analysis, using Fischer’s Exact test and the Mann-Whitney U test, were carried out to determine associations between clinicopathological parameters and colorectal cancer-specific mortality after four years. Disease-specific survival curves were formed using the Kaplan-Meier method. The Cox proportional-hazards model was used to estimate the hazard ratio (HR) and its associated confidence intervals to determine the extent and significance of associations between lymphatic invasion detected by either H&E or D2-40 staining with disease-specific survival. p-Values less than 0.05 were considered statistically significant. 

## Results

**Figure 1:**
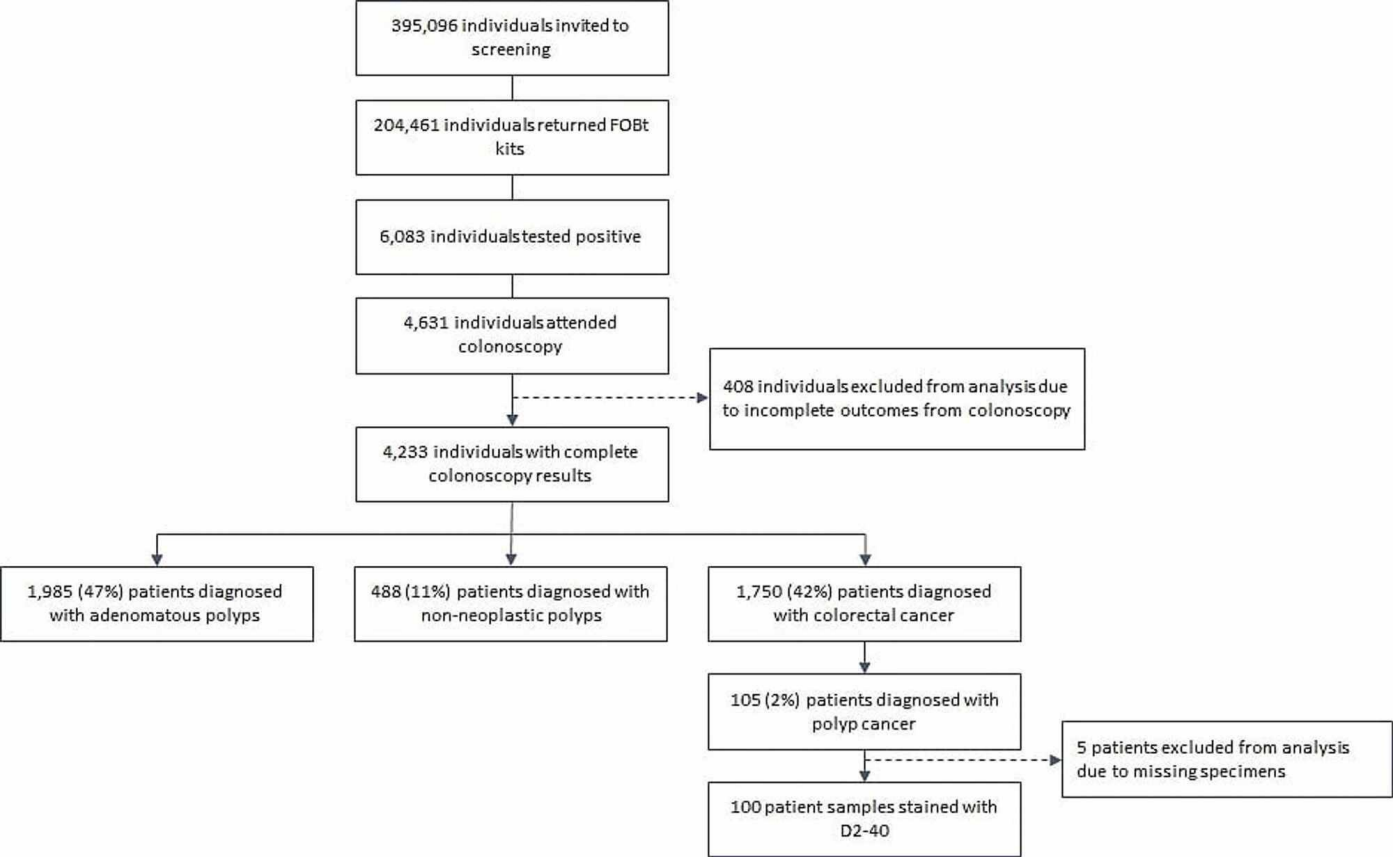
Patient selection from the Greater Glasgow & Clyde Bowel Screening Registry.

The Greater Glasgow and Clyde Bowel Cancer Screening Registry contained a total of 395,096 individuals who were invited to the first round of FOBt screening. Over 6,083 individuals returned a positive FOBt and were invited to colonoscopy. Of these, 4,631 attended follow-up colonoscopy and 105 patients had pathologically diagnosed colorectal polyp cancer and hence met the inclusion criteria. Five patients were excluded due to incomplete or missing specimens resulting in a final sample size of 100. The patient selection method is outlined in Figure 1.

The 100 patients included within the study consisted of 68 males and 32 females with a median age at polypectomy of 66 years (range 50-76 years). Forty-six patients from this cohort underwent further resectional surgery after polypectomy. At the time of diagnosis, no patients had any evidence of distant or nodal metastasis determined by staging CT scanning or further resectional surgery. 

Over 170 polypectomy specimens from this cohort of patients were found to contain colorectal polyp cancers and each patient had between one and three cancer-containing paraffin-embedded specimens. Of these 170 specimens, nine (5%) were sessile lesions removed by endoscopic mucosal resection and 161 (95%) were polypoid lesion removed by snare polypectomy. 

**Figure 2:**
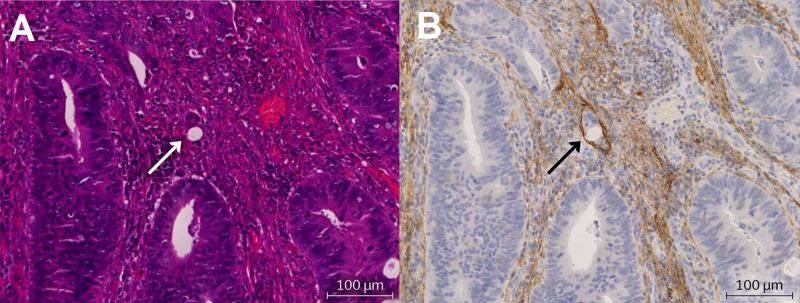
An example of lymphatic invasion within matched specimens stained with hematoxylin & eosin (H&E) (A) and D2-40 (B). (A) H&E staining and (B) D2-40 staining.

Immunoreactivity with the D2-40 antibody was detectable in all stained specimens. Figure 2B is an example of D2-40 immunostaining highlighting a lymphatic channel that contains a nest of tumour cells - allowing the diagnosis of lymphatic invasion. In contrast, it is almost impossible to recognise that the tumour cells are contained within a lymphatic vessel in the matched H&E-stained slide, shown in Figure 2A.

The lymphatic invasion was reported in the pathology reports of eight patients (8%) using H&E staining. Whereas, D2-40 immunohistochemistry detected lymphatic invasion in 23 patients (23%). McNemar’s test demonstrated a significant difference between the proportions of lymphatic invasion detected by D2-40 or H&E staining (p=0.01) with D2 40 staining detecting lymphatic invasion in 15.0% (95% CI 14.1-15.9%) more patients than H&E staining. Positive lymphatic invasion on both stains was only detected in three (3%) patients with a disagreement between H&E and D2-40 staining in 25 (25%) patients (Table 1).

**Table 1:** Detection of lymphatic invasion using H&E versus D2-40 staining. p-value = 0.004 (value based on McNemar’s test).

		Detected by D2-40	
		Lymphatic invasion negative	Lymphatic invasion positive
Detected by H&E	Lymphatic invasion negative	72	20
	Lymphatic invasion positive	5	3

The minimum follow-up time for patients was four years. During this time, 6 out of 100 patients died, with five deaths occurring due to colorectal cancer. Table 2 shows the clinical and pathological variables included in this study and their relationship with colorectal cancer-specific mortality after four years using univariate analysis. No clinical or pathological variables other than D2-40 detected lymphatic invasion demonstrated a statistically significant relationship with colorectal cancer-specific mortality after four years. A p-value of 0.01 was calculated by Fischer’s exact test for the association between D2-40-detected lymphatic invasion and four-year colorectal cancer-specific survival.

**Table 2:** Association of clinicopathological parameters with colorectal cancer-specific mortality four years after polypectomy. ^a ^Value based on the Mann-Whitney U test; ^b^ Value based on Fischer’s exact test.

	Colorectal cancer-specific mortality after five years		Univariate p-value
	Alive or dead due to other causes (n=95)	Dead due to colorectal cancer (n=5)	
Age	66.07 (±6.84)	67.00 (±10.30)	0.54^a^
Gender			
Male	66	2	0.32^b^
Female	29	3	
Further resectional surgery			
Yes	43	3	0.66^b^
No	52	2	
Tumour location			
Colon	71	3	0.60^b^
Rectum	24	2	
Tumour differentiation			
Well	91	5	1.00^b^
Moderate or poor	4	0	
Venous invasion			
Positive	21	2	0.32^b^
Negative	74	3	
Lymphatic Invasion			
H&E detected	8	0	1.00^b^
D2-40 detected	19	4	0.01^b^

**Figure 3:**
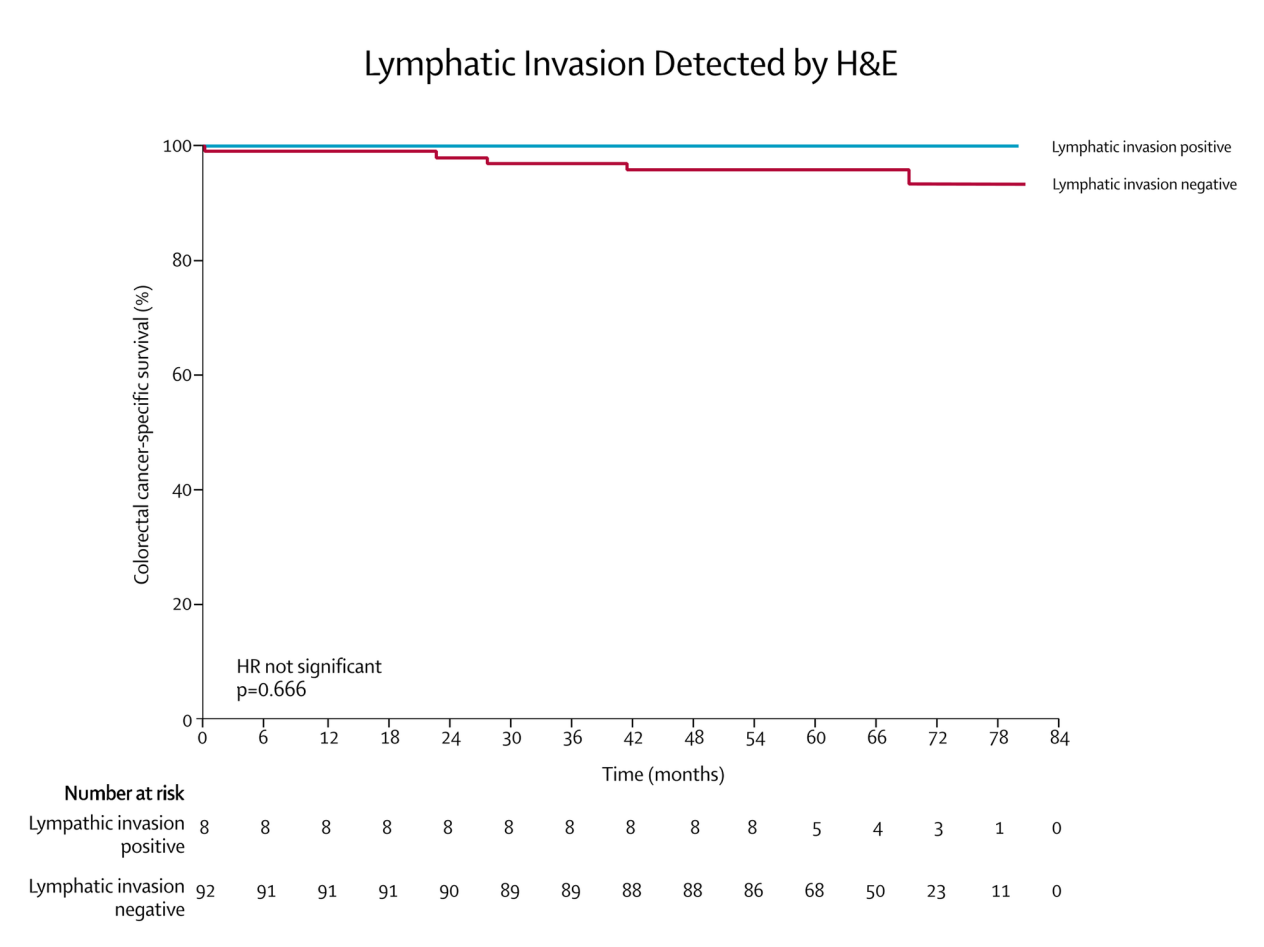
Kaplan-Meier survival analysis of colorectal cancer-specific survival depending on H&E-detected lymphatic invasion. p-values and hazard ratios calculated by Cox regression.

**Figure 4:**
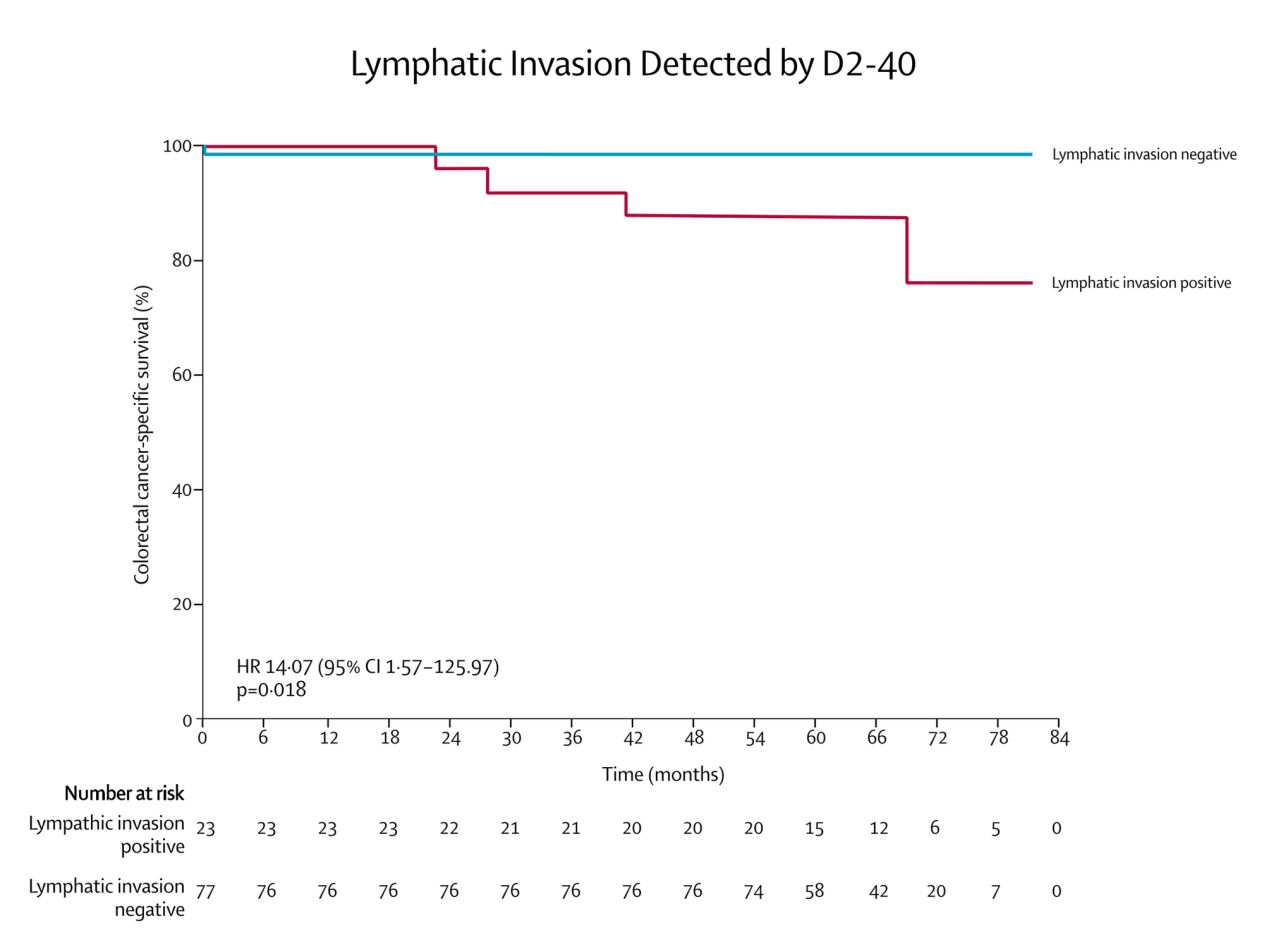
Kaplan-Meier survival analysis of colorectal cancer-specific survival depending on D2-40-detected lymphatic invasion. p-values and hazard ratios calculated by Cox regression.

Survival analysis performed separately by Cox regression for D2-40- and H&E-detected lymphatic invasion demonstrated that only lymphatic invasion detected by D2-40 was significantly associated with an increase in the risk of colorectal cancer death (HR 14.07, 95% CI 1.57-125.97, p=0.018). The Kaplan-Meier curves for H&E and D2-40 detected lymphatic invasion and are shown in Figures 3 and 4.

## Discussion

Lymphatic invasion is an important prognostic indicator in colorectal polyp cancer and a determinant of the extent of treatment required [26]. Nevertheless, the current pathological diagnosis of lymphatic invasion can be easily missed or influenced by the global image of the tumour when using H&E staining alone [10]. The findings of this study demonstrate that the detection of lymphatic invasion can be significantly improved by using D2-40 immunostaining compared to H&E staining. In addition, D2-40-detected lymphatic invasion was found to have prognostic significance, unlike all other clinicopathological variables which were investigated, including H&E-detected lymphatic invasion.

In the present study, the incidence rate of lymphatic invasion in colorectal polyp cancer was 8% using H&E staining and 23% using D2-40 immunohistochemistry. The 15% absolute increase in detection rates found is similar to previously reported uses of D2-40 in other stages of colorectal cancer [16,20,27]. D2-40 immunostaining provides a reliable diagnosis of lymphatic invasion and as such the authors are confident that any lymphatic invasion detected by this method represents true-positives [18,19]. In this study, 20% of patients had lymphatic invasion detected on D2-40 immunostaining but were falsely reported as having no lymphatic invasion with H&E staining. The high incidence rate of false-negatives with H&E staining prevents lymphatic invasion detected by this method from having any prognostic significance as demonstrated by the results in this study and other research [25]. More importantly, these findings also reveal that underdiagnosis of lymphatic invasion is a common problem in colorectal polyp cancer.

In contrast, false-positive reporting of lymphatic invasion can also occur and is a well-reported phenomenon estimated to account for up to 68% of all H&E-detected lymphatic invasion in colorectal cancer [28]. Many structures within adenocarcinomas, under H&E staining, can resemble lymphatic invasion and result in false-positive reporting. These structures include invasion of tumour cells into capillary channels, peritumoural or intratumoural carcinomatous vesiculous lesions, and retraction artefact around cancer nests [29]. Five percent of patients within this study were reported as having lymphatic invasion on H&E staining but were not detected by D2-40 immunostaining; most likely representing false positive reporting associated with H&E staining. However, this cannot be confirmed as inspection of the H&E stained specimens was not performed alongside D2-40 staining in this study. Instead, H&E-detected lymphatic invasion was determined by pathology reports carried out at the time of diagnosis by various pathologists. This introduces a potential source of variability as reporting was performed by multiple individuals. 

In reality, this variability is likely to be negligible as all reporting was performed by consultant pathologists with similar levels of experience. More importantly, this study design prevented the side-by-side inspection of D2-40 and H&E stained slides which would have allowed confirmation of any false-reporting. Nevertheless, this study design has the benefit of allowing a direct comparison between the novel D2-40 staining procedure to current clinical practiced. In addition, to the limitations discussed above, two other limitations exist which could have prevented lymphatic invasion from being detected on D2-40 staining but allowed detection on diagnostic H&E staining meaning that the lack of agreement between the staining procedures may not completely represent false-reporting on H&E staining. 

The first limitation is related to the D2-40 immunohistochemistry technique as a small proportion of specimens suffered from high background staining potentially preventing accurate identification of lymphatic invasion. It is unlikely that the staining procedure itself accounted for this variation in staining quality as stringent optimisation and control procedures were performed. In addition, other specimens stained within the same batch as those affected by high background staining had very little background staining. These factors, together, indicate that differences within the archived specimens themselves such as age, storage, and formalin-fixing and paraffin-embedding procedures could have resulted in this variability in staining. Problems with high background staining should not prevent the routine use of D2-40 in clinical practice as they are unlikely to be significant when the immunohistochemistry technique is optimised for new clinical samples that have undergone standardised embedding and fixing procedures.

Second, the specimens used for D2-40 immunostaining within this study contained less tumour volume than when originally used for diagnostic reporting as multiple tissue cores had been removed from almost all patient specimens to create tumour microarrays (TMA) for use in another research project. This could have potentially removed areas containing lymphatic invasion which had been previously detected on H&E staining. Although, it can only be suggested that lack of tumour volume may have resulted in reduced detection of lymphatic invasion in our D2-40 staining slides, the importance of having adequate tumour volume for objective reporting of lymphatic invasion has been highlighted. In the future, the authors recommend that the absolute number of lymphatic channels or total tumour volume present in slides be investigated with the aim of assessing the minimum required to allow negative reporting. Any specimens under this threshold can be reported as inconclusive rather than negative in a manner similar to breast cytology reporting that requires a minimum of at least six benign cell groups before allowing negative diagnosis [30]. 

Although the survival relationship shown with D2-40-detected lymphatic invasion was statistically significant, the small sample size coupled with the low mortality of this disease means that the HR could not be estimated accurately making the clinical significance of the relationship between lymphatic invasion and survival difficult to determine. In addition, multivariate statistical analysis could not be performed due to an insufficient number of events (disease-specific death) within this cohort. Although tested with univariate analysis, confounding factors, such as further resectional surgery, vascular invasion and tumor differentiation cannot be ruled out until a multivariable model is formed to consider each of these as well as D2-40-detected lymphatic invasion.

## Conclusions

This study has importantly been able to demonstrate that lymphatic invasion detected by D2-40 immunostaining is significantly associated with reduced survival in screening-detected colorectal polyp cancer. This study has been able to demonstrate for the first time that the improved detection and prognostic significance of D2-40-detected lymphatic invasion shown in studies of advanced colorectal cancer still hold true for polyp cancer. These results indicate that the routine clinical use of D2-40 immunostaining in colorectal polyp cancer is justified as it may allow increased detection of lymphatic invasion, improved risk-stratification of patients, and better clinical decision making. 
